# The genome sequence of the Dark Arches *Apamea monoglypha* (Hufnagel, 1766)

**DOI:** 10.12688/wellcomeopenres.18947.1

**Published:** 2023-02-08

**Authors:** Douglas Boyes, University of Oxford and Wytham Woods Genome Acquisition Lab, Darwin Tree of Life Barcoding collective, Wellcome Sanger Institute Tree of Life programme, Wellcome Sanger Institute Scientific Operations: DNA Pipelines collective, Tree of Life Core Informatics collective, John. F. Mulley, Darwin Tree of Life Consortium

**Affiliations:** 1 UK Centre for Ecology and Hydrology, Wallingford, Oxfordshire, UK; 2 School of Natural Sciences, Bangor University, Bangor, Wales, UK

**Keywords:** Apamea monoglypha, Dark Arches, genome sequence, chromosomal, Lepidoptera

## Abstract

We present a genome assembly from an individual male *Apamea monoglypha* (the Dark Arches, Arthropoda; Insecta; Lepidoptera; Noctuidae). The genome sequence is 576 megabases in span. Most of the assembly is scaffolded into 31 chromosomal pseudomolecules, including the assembled Z sex chromosome. The mitochondrial genome has also been assembled and is 16.5 kilobases in length. Gene annotation of this assembly on Ensembl has identified 17,963 protein coding genes.

## Species taxonomy

Eukaryota; Metazoa; Ecdysozoa; Arthropoda; Hexapoda; Insecta; Pterygota; Neoptera; Endopterygota; Lepidoptera; Glossata; Ditrysia; Noctuoidea; Noctuidae; Noctuinae; Apameini; *Apamea*; *Apamea monoglypha* (Hufnagel, 1766) (NCBI:txid875885).

## Background

The Dark Arches *Apamea monoglypha* Hufnagel, 1766 is a large (45–55 mm wingspan) noctuid moth that is common in Europe and has scattered records from elsewhere across the western Palearctic. It can be extremely abundant in some locations in the south of the UK. A recent review of macro-moth status classified *A. monoglypha* as being widespread and abundant in Great Britain and placed it in the ‘Least Concern’ IUCN Red List category ( Fox *et al.*, 2019). Adults are primarily on the wing from June to September, with a peak abundance in July; the larvae feed on a range of grasses before overwintering among the bases and roots of these plants. There is sometimes a second brood later in the year in the more southern parts of the UK ( Knill-Jones, 2005).

*A. monoglypha* is easily recognised by its size, distinct oval and kidney markings, and a ‘W’-shaped line at the outer edge (termen) of the forewings; overall colouration is variable, with specimens ranging from a light cream colour through to almost fully black. A melanic form (f. *aethiops* ( Tutt, 1891)) has been recorded, which lacks the typical markings. Kettlewell considered the melanic form to be an example of ancient and non-industrial melanism (albeit with localised incidences of industrial melanism) under the control of a single locus with the melanic form dominant ( Kettlewell, 1973), although possibly with influence from other genetic or environmental factors ( Bishop *et al.,* 1976). The more variable background colouration is likely polygenic ( Cockayne, 1938; Fraiers *et al.*, 1994).

A genome assembly for *Apamea monoglypha* will be invaluable in identifying the genetic basis of colour polymorphism in this species and facilitate further research into this often abundant and ecologically important species.

### Genome sequence report

**Figure 1.**
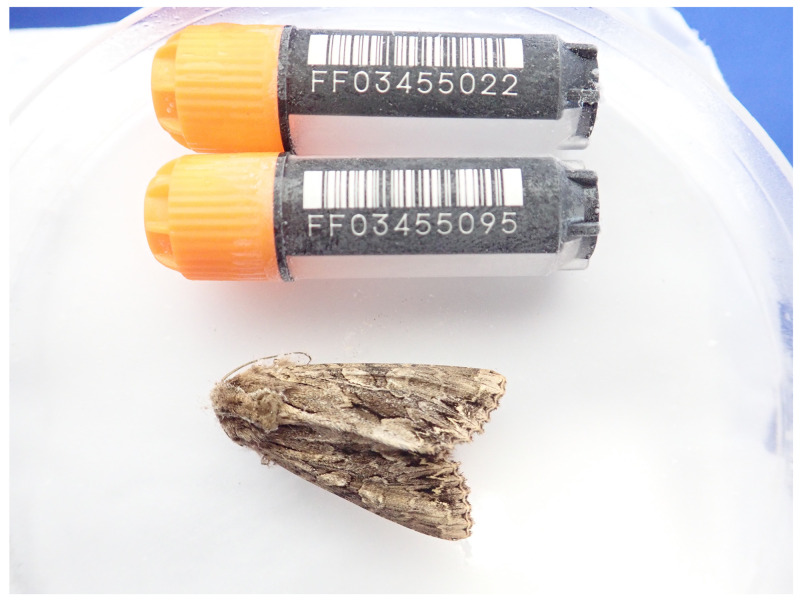
Photograph of the *Apamea monoglypha* (ilApaMono1) specimen used for genome sequencing.

The genome was sequenced from one male *A. monoglypha* specimen ( Figure 1) collected from Wytham Woods, UK (latitude 51.77, longitude –1.34). A total of 25-fold coverage in Pacific Biosciences single-molecule HiFi long reads and 60-fold coverage in 10X Genomics read clouds were generated. Primary assembly contigs were scaffolded with chromosome conformation Hi-C data. Manual assembly curation corrected 52 missing joins or mis-joins and removed 14 haplotypic duplications, reducing the assembly length by 1.02% and the scaffold number by 54.79%, and increasing the scaffold N50 by 7.26%.

**Figure 2.**
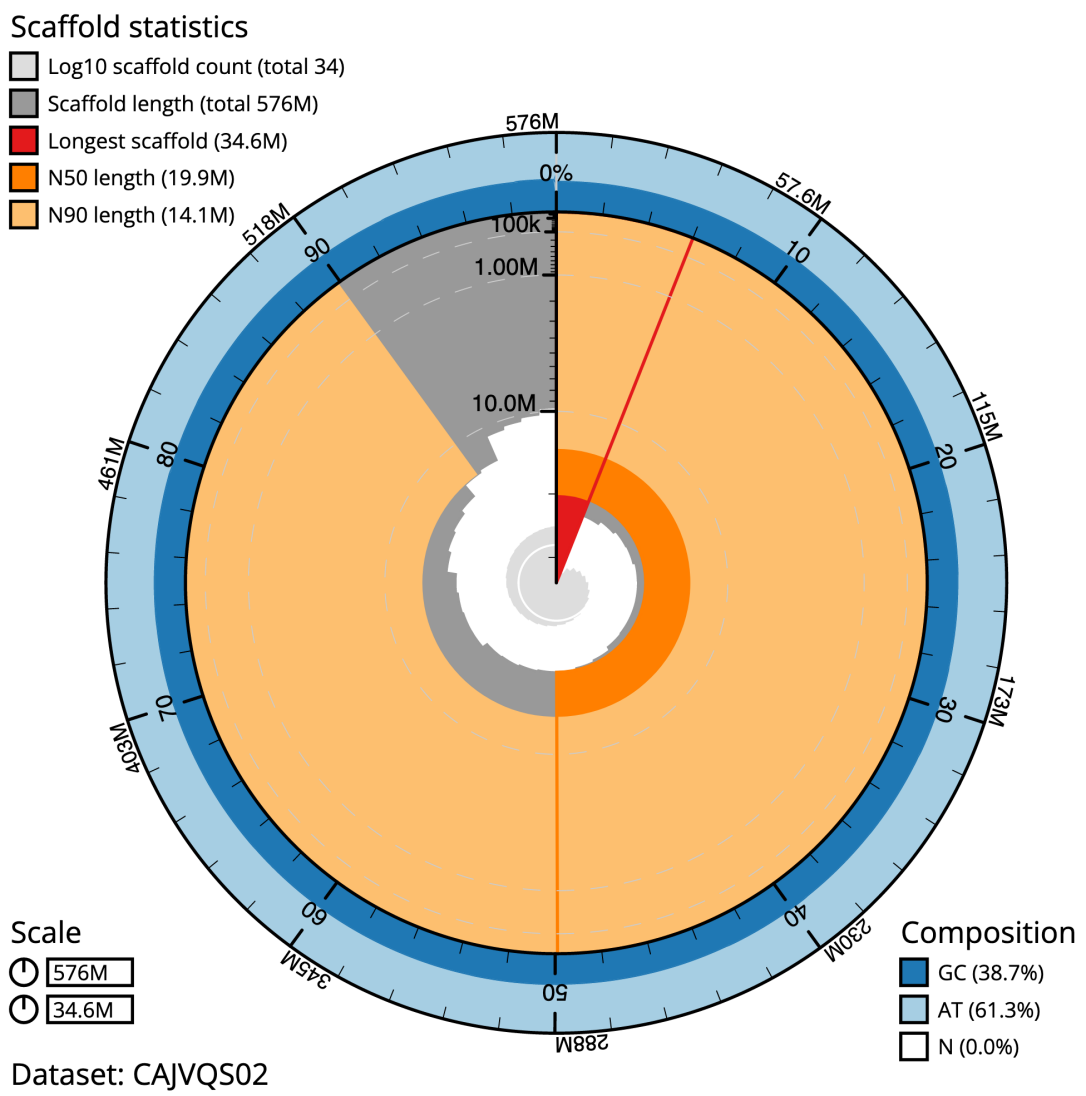
Genome assembly of *Apamea monoglypha*, ilApaMono1.2: metrics. The BlobToolKit Snailplot shows N50 metrics and BUSCO gene completeness. The main plot is divided into 1,000 size-ordered bins around the circumference with each bin representing 0.1% of the 575,683,298 bp assembly. The distribution of scaffold lengths is shown in dark grey with the plot radius scaled to the longest scaffold present in the assembly (34,587,019 bp, shown in red). Orange and pale-orange arcs show the N50 and N90 scaffold lengths (19,918,846 and 14,112,846 bp), respectively. The pale grey spiral shows the cumulative scaffold count on a log scale with white scale lines showing successive orders of magnitude. The blue and pale-blue area around the outside of the plot shows the distribution of GC, AT and N percentages in the same bins as the inner plot. A summary of complete, fragmented, duplicated and missing BUSCO genes in the lepidoptera_odb10 set is shown in the top right. An interactive version of this figure is available at https://blobtoolkit.genomehubs.org/view/ilApaMono1.2/dataset/CAJVQS02/snail.

The final assembly has a total length of 575.7 Mb in 33 sequence scaffolds with a scaffold N50 of 19.9 Mb ( Table 1). Most (99.99%) of the assembly sequence was assigned to 31 chromosomal-level scaffolds, representing 30 autosomes and the Z sex chromosome. Chromosome-scale scaffolds confirmed by the Hi-C data are named in order of size ( Figure 2– Figure 5; Table 2). The assembly has a BUSCO v5.3.2 ( Manni *et al.*, 2021) completeness of 99.0% using the lepidoptera_odb10 reference set. While not fully phased, the assembly deposited is of one haplotype. Contigs corresponding to the second haplotype have also been deposited.

**Table 1.** Genome data for *Apamea monoglypha*, ilApaMono1.2.

Project accession data		
Assembly identifier	ilApaMono1.2	
Species	*Apamea monoglypha*	
Specimen	ilApaMono1	
NCBI taxonomy ID	875885	
BioProject	PRJEB45191	
BioSample ID	SAMEA7701555	
Isolate information		
Assembly metrics *		*Benchmark*
Consensus quality (QV)	59.7	*≥ 50*
*k*-mer completeness	100%	*≥ 95%*
BUSCO **	C:99.0%[S:98.2%,D:0.8%], F:0.3%,M:0.7%,n:5,286	*C ≥ 95%*
Percentage of assembly mapped to chromosomes	99.99%	*≥ 95%*
Sex chromosomes	Z chromosome	*localised homologous pairs*
Organelles	Mitochondrial genome assembled	*complete single alleles*
**Raw data accessions**		
PacificBiosciences SEQUEL II	ERR6436385	
10X Genomics Illumina	ERR6054941–ERR6054944	
Hi-C Illumina	ERR6054940	
Genome assembly		
Assembly accession	GCA_911387735.2	
*Accession of alternate haplotype*	GCA_911387795.2	
Span (Mb)	575.7	
Number of contigs	107	
Contig N50 length (Mb)	9.1	
Number of scaffolds	33	
Scaffold N50 length (Mb)	19.9	
Longest scaffold (Mb)	34.6	
Genome annotation		
Number of protein-coding genes	17,963	
Number of gene transcripts	18,157	

* Assembly metric benchmarks are adapted from column VGP-2020 of “ Table 1: Proposed standards and metrics for defining genome assembly quality” from ( Rhie *et al.*, 2021). ** BUSCO scores based on the lepidoptera_odb10 BUSCO set using v5.3.2. C = complete [S = single copy, D = duplicated], F = fragmented, M = missing, n = number of orthologues in comparison. A full set of BUSCO scores is available at https://blobtoolkit.genomehubs.org/view/ilApaMono1.2/dataset/CAJVQS02/busco.

**Figure 3.**
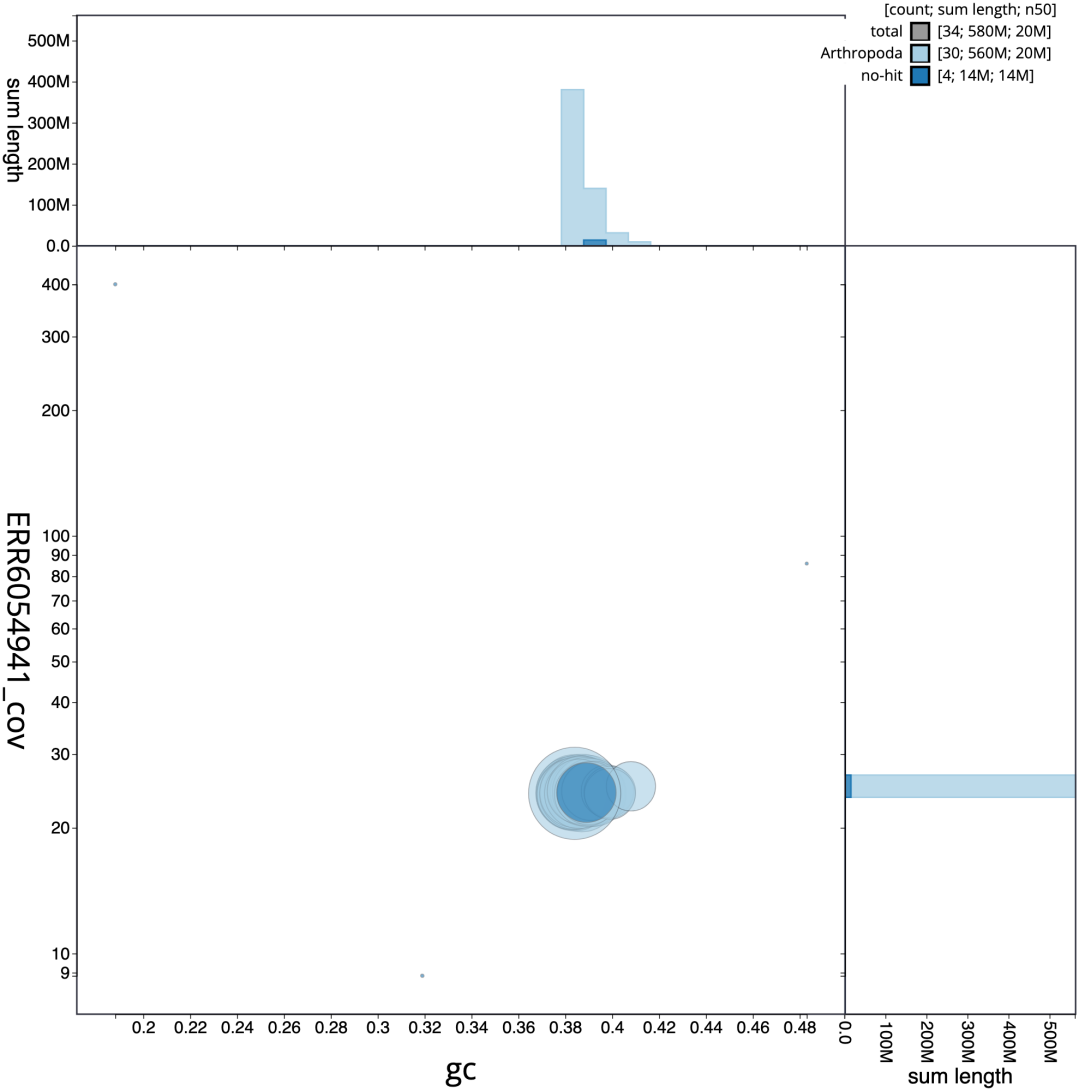
Genome assembly of *Apamea monoglypha*, ilApaMono1.2: GC coverage. BlobToolKit GC-coverage plot. Scaffolds are coloured by phylum. Circles are sized in proportion to scaffold length. Histograms show the distribution of scaffold length sum along each axis. An interactive version of this figure is available at https://blobtoolkit.genomehubs.org/view/ilApaMono1.2/dataset/CAJVQS02/blob.

**Figure 4.**
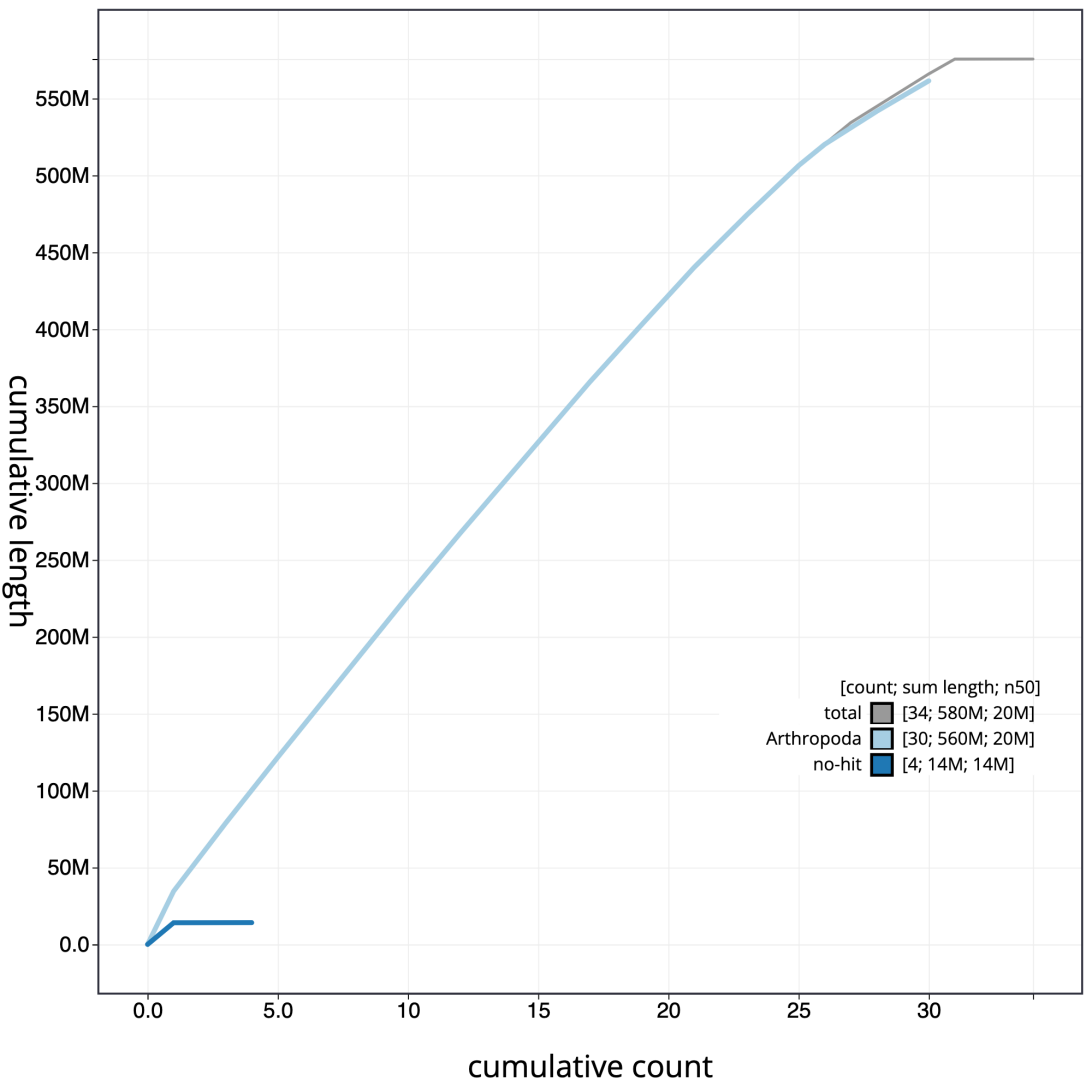
Genome assembly of *Apamea monoglypha*, ilApaMono1.2: cumulative sequence. BlobToolKit cumulative sequence plot. The grey line shows cumulative length for all scaffolds. Coloured lines show cumulative lengths of scaffolds assigned to each phylum using the buscogenes taxrule. An interactive version of this figure is available at https://blobtoolkit.genomehubs.org/view/ilApaMono1.2/dataset/CAJVQS02/cumulative.

**Figure 5.**
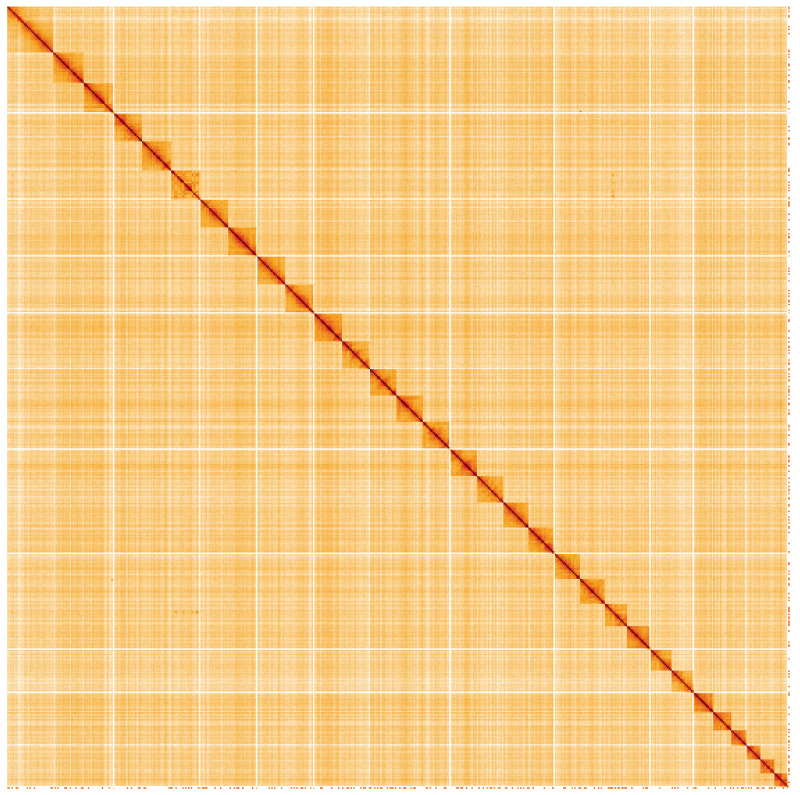
Genome assembly of *Apamea monoglypha*, ilApaMono1.2: Hi-C contact map. Hi-C contact map of the ilApaMono1.2 assembly, visualised using HiGlass. Chromosomes are shown in order of size from left to right and top to bottom. An interactive version of this figure may be viewed at https://genome-note-higlass.tol.sanger.ac.uk/l/?d=QFTmrUfDS3i4DilDoNn-Cw.

**Table 2.** Chromosomal pseudomolecules in the genome assembly of *Apamea monoglypha*, ilApaMono1.

INSDC accession	Chromosome	Size (Mb)	GC%
OU426915.1	1	22.42	38.4
OU426916.1	2	21.85	38.7
OU426917.1	3	21.47	38.3
OU426918.1	4	21.36	38.3
OU426919.1	5	21.06	38.7
OU426920.1	6	21.03	38.4
OU426921.1	7	21.01	38.9
OU426922.1	8	21.01	38.5
OU426923.1	9	20.92	38.3
OU426924.1	10	20.41	38.7
OU426925.1	11	20.17	38.3
OU426926.1	12	19.96	38.3
OU426927.1	13	19.92	38.6
OU426928.1	14	19.77	38.4
OU426929.1	15	19.59	38.4
OU426930.1	16	19.48	38.6
OU426931.1	17	18.74	38.8
OU426932.1	18	18.72	38.7
OU426933.1	19	18.57	38.9
OU426934.1	20	18.33	39.1
OU426935.1	21	17.07	39.2
OU426936.1	22	16.66	38.6
OU426937.1	23	16.38	38.9
OU426938.1	24	15.87	39
OU426939.1	25	14.11	38.9
OU426940.1	26	13.81	39.1
OU426941.1	27	10.92	39.8
OU426942.1	28	10.59	39.9
OU426943.1	29	10.23	39.9
OU426944.1	30	9.61	40.8
OU426914.1	Z	34.59	38.4
OU426945.1	MT	0.02	19
-	unplaced	0.03	38.6

### Genome annotation report

The *A. monoglypha* GCA_911387795.2 genome assembly was annotated using the Ensembl rapid annotation pipeline ( Table 1; https://rapid.ensembl.org/Apamea_monoglypha_GCA_911387795.2/). The resulting annotation includes 18,157 transcribed mRNAs from 17,963 protein-coding genes.

## Methods

### Sample acquisition and nucleic acid extraction

One *A. monoglypha* specimen (ilApaMono1) was collected in Wytham Woods, Oxfordshire (biological vice-county: Berkshire), UK (latitude 51.77, longitude –1.34) on 20 July 2020 using a light trap. The specimen was collected and identified by Douglas Boyes (University of Oxford) and snap-frozen on dry ice.

DNA was extracted at the Tree of Life laboratory, Wellcome Sanger Institute (WSI). The ilApaMono1 sample was weighed and dissected on dry ice with tissue set aside for Hi-C sequencing. Abdomen tissue was cryogenically disrupted to a fine powder using a Covaris cryoPREP Automated Dry Pulveriser, receiving multiple impacts. High molecular weight (HMW) DNA was extracted using the Qiagen MagAttract HMW DNA extraction kit. Low molecular weight DNA was removed from a 20 ng aliquot of extracted DNA using 0.8X AMpure XP purification kit prior to 10X Chromium sequencing; a minimum of 50 ng DNA was submitted for 10X sequencing. HMW DNA was sheared into an average fragment size of 12–20 kb in a Megaruptor 3 system with speed setting 30. Sheared DNA was purified by solid-phase reversible immobilisation using AMPure PB beads with a 1.8X ratio of beads to sample to remove the shorter fragments and concentrate the DNA sample. The concentration of the sheared and purified DNA was assessed using a Nanodrop spectrophotometer and Qubit Fluorometer and Qubit dsDNA High Sensitivity Assay kit. Fragment size distribution was evaluated by running the sample on the FemtoPulse system.

### Sequencing

Pacific Biosciences HiFi circular consensus and 10X Genomics read cloud DNA sequencing libraries were constructed according to the manufacturers’ instructions. DNA sequencing was performed by the Scientific Operations core at the WSI on Pacific Biosciences SEQUEL II (HiFi) and Illumina NovaSeq 6000 (10X) instruments. Hi-C data were also generated from head and thorax tissue of ilApaMono1 using the Arima v2 kit and sequenced on the Illumina NovaSeq 6000 instrument.

### Genome assembly

Assembly was carried out with Hifiasm ( Cheng *et al.*, 2021) and haplotypic duplication was identified and removed with purge_dups ( Guan *et al.*, 2020). One round of polishing was performed by aligning 10X Genomics read data to the assembly with Long Ranger ALIGN, calling variants with freebayes ( Garrison & Marth, 2012). The assembly was then scaffolded with Hi-C data ( Rao *et al.*, 2014) using SALSA2 ( Ghurye *et al.*, 2019). The assembly was checked for contamination and corrected using the gEVAL system ( Chow *et al.*, 2016) as described previously ( Howe *et al.*, 2021). Manual curation was performed using gEVAL, HiGlass ( Kerpedjiev *et al.*, 2018) and Pretext ( Harry, 2022). The mitochondrial genome was assembled using MitoHiFi ( Uliano-Silva *et al.*, 2022), which performed annotation using MitoFinder ( Allio *et al.*, 2020). The genome was analysed and BUSCO scores generated within the BlobToolKit environment ( Challis *et al.*, 2020). Table 3 contains a list of all software tool versions used, where appropriate.

**Table 3.** Software tools and versions used.

Software tool	Version	Source
BlobToolKit	3.5.0	Challis *et al.*, 2020
freebayes	1.3.1-17- gaa2ace8	Garrison & Marth, 2012
gEVAL	N/A	Chow *et al.*, 2016
Hifiasm	0.14-r312	Cheng *et al.*, 2021
HiGlass	1.11.6	Kerpedjiev *et al.*, 2018
Long Ranger ALIGN	2.2.2	https://support.10xgenomics.com/ genome-exome/software/pipelines/ latest/advanced/other-pipelines
MitoHiFi	2.11	Uliano-Silva *et al.*, 2022
PretextView	0.2	Harry, 2022
purge_dups	1.2.3	Guan *et al.*, 2020
SALSA	2.2	Ghurye *et al.*, 2019

### Genome annotation

The BRAKER2 pipeline ( Brůna *et al.*, 2021) was used in the default protein mode to generate annotation for the *Apamea monoglypha* assembly (GCA_911387795.2) in Ensembl Rapid Release.

### Ethics/compliance issues

The materials that have contributed to this genome note have been supplied by a Darwin Tree of Life Partner. The submission of materials by a Darwin Tree of Life Partner is subject to the Darwin Tree of Life Project Sampling Code of Practice. By agreeing with and signing up to the Sampling Code of Practice, the Darwin Tree of Life Partner agrees they will meet the legal and ethical requirements and standards set out within this document in respect of all samples acquired for, and supplied to, the Darwin Tree of Life Project. Each transfer of samples is further undertaken according to a Research Collaboration Agreement or Material Transfer Agreement entered into by the Darwin Tree of Life Partner, Genome Research Limited (operating as the Wellcome Sanger Institute), and in some circumstances other Darwin Tree of Life collaborators.

## Data Availability

European Nucleotide Archive: *Apamea monoglypha* (dark arches). Accession number PRJEB45191; https://identifiers.org/ena.embl/PRJEB45191. ( Wellcome Sanger Institute, 2021) The genome sequence is released openly for reuse. The *Apamea monoglypha* genome sequencing initiative is part of the Darwin Tree of Life (DToL) project. All raw sequence data and the assembly have been deposited in INSDC databases. Raw data and assembly accession identifiers are reported in Table 1. Members of the University of Oxford and Wytham Woods Genome Acquisition Lab are listed here: https://doi.org/10.5281/zenodo.4789928. Members of the Darwin Tree of Life Barcoding collective are listed here: https://doi.org/10.5281/zenodo.4893703. Members of the Wellcome Sanger Institute Tree of Life programme are listed here: https://doi.org/10.5281/zenodo.4783585. Members of Wellcome Sanger Institute Scientific Operations: DNA Pipelines collective are listed here: https://doi.org/10.5281/zenodo.4790455. Members of the Tree of Life Core Informatics collective are listed here: https://doi.org/10.5281/zenodo.5013541. Members of the Darwin Tree of Life Consortium are listed here: https://doi.org/10.5281/zenodo.4783558.
