# Supplementary material for: The genome sequence of the Dark Arches Apamea monoglypha (Hufnagel, 1766)
Source: Wellcome Open Res. 2023 Feb 8;8:63. [Version 1] doi: 10.12688/wellcomeopenres.18947.1 (PMC11316173; doi:10.12688/wellcomeopenres.18947.1)
Supplement: Peer review history

# Reviewer report: Reviewer response for version 1

**DOI:** 10.21956/wellcomeopenres.21007.r64634

**Published:** 2024-08-09

**Authors:** Min-jin Han

**Affiliations:** 1 State Key Laboratory of Resource Insects, Institute of Sericulture and Systems Biology, Southwest University, BeiBei, Chongqing, China

I greatly admire the authors for completing the high-quality genome of this species. It would be even better if there could be improvements in the annotation of genomic repetitive sequences and the annotation and quality assessment of protein-coding genes. Additionally, most moths exhibit female heterochromosomes. If the genome of female individuals could be sequenced as much as possible, it would be more representative.

Are sufficient details of methods and materials provided to allow replication by others?

Partly

Is the rationale for creating the dataset(s) clearly described?

Yes

Are the datasets clearly presented in a useable and accessible format?

Yes

Are the protocols appropriate and is the work technically sound?

Partly

Reviewer Expertise:

Genomics;Genetics and Evolution; Insects

I confirm that I have read this submission and believe that I have an appropriate level of expertise to confirm that it is of an acceptable scientific standard.

# Reviewer report: Reviewer response for version 1

**DOI:** 10.21956/wellcomeopenres.21007.r87697

**Published:** 2024-07-04

**Authors:** Hongmei Li-Byarlay

**Affiliations:** 1 Central State University, Wilberforce, Ohio, USA

This data note titled "The genome sequence of the Dark Arches *Apamea* *monoglypha* (Hufnagel, 1766)" aligns with the objectives proposed by the Darwin Tree of Life Consortium and consequently by the Earth Biogenome Project. This genome project aimed to enrich our current knowledge in genetics and conservation. It provided unique information about colour polymorphism, evolution, and ecology.

 The rationale for creating the genomic data is clearly described. The protocols are appropriate and provide adequate information and detail of the technical procedure allowing others to repeat it.

 The dataset is presented in a valid and accessible format, and deposited in the NCBI database.

 For genetics, a female moth has a heterogametic sex chromosome system, with most females having a WZ constitution. The males are ZZ chromosome system. Is there a plan to sequence a female sample to capture the full WZ constitution? It will be great to provide the genetic information of this species in the introduction and point out how the male genomic sequence plays an role in studying this species or macro-moth species.

Are sufficient details of methods and materials provided to allow replication by others?

Yes

Is the rationale for creating the dataset(s) clearly described?

Yes

Are the datasets clearly presented in a useable and accessible format?

Yes

Are the protocols appropriate and is the work technically sound?

Yes

Reviewer Expertise:

Entomology, Genomics,  Molecular Biology, Behavioral Ecology

I confirm that I have read this submission and believe that I have an appropriate level of expertise to confirm that it is of an acceptable scientific standard.
